# Acupuncture Relieved Chemotherapy-Induced Peripheral Neuropathy in Patients with Breast Cancer: A Pilot Randomized Sham-Controlled Trial

**DOI:** 10.3390/jcm10163694

**Published:** 2021-08-20

**Authors:** Chien-Chen Huang, Tsung-Jung Ho, Hsin-Yueh Ho, Pei-Yu Chen, Cheng-Hao Tu, Yu-Chuen Huang, Yu-Chen Lee, Mao-Feng Sun, Yi-Hung Chen

**Affiliations:** 1 Graduate Institute of Chinese Medicine, School of Chinese Medicine, College of Chinese Medicine, China Medical University, Taichung 40402, Taiwan; chenchinh2013@gmail.com (C.-C.H.); d5167@mail.cmuh.org.tw (Y.-C.L.); 2 Department of Traditional Chinese Medicine, An Nan Hospital, China Medical University, Tainan City 709, Taiwan; 3 Department of Chinese Medicine, Hualien Tzu Chi Hospital, Hualien 970, Taiwan; jeron888@gmail.com; 4 School of Post-Baccalaureate Chinese Medicine, Tzu Chi University, Hualien 970374, Taiwan; 5 Integration Center of Traditional Chinese and Modern Medicine, Hualien Tzu Chi Hospital, Hualien 970, Taiwan; 6 Department of Surgery, An Nan Hospital, China Medical University, Tainan City 709, Taiwan; n71721@mail.tmanh.org.tw; 7 Cancer Center, An Nan Hospital, China Medical University, Tainan City 709, Taiwan; n73362@mail.tmanh.org.tw; 8 Graduate Institute of Acupuncture Science, China Medical University, Taichung 40402, Taiwan; lordowentu@gmail.com (C.-H.T.); maofeng@mail.cmuh.org.tw (M.-F.S.); 9 Department of Medical Research, China Medical University Hospital, School of Chinese Medicine, China Medical University, Taichung 40402, Taiwan; yuchuen@mail.cmu.edu.tw; 10 Department of Acupuncture, China Medical University Hospital, Taichung 404332, Taiwan; 11 Chinese Medicine Research Center, China Medical University, Taichung 40402, Taiwan; 12 Department of Photonics and Communication Engineering, Asia University, Taichung 41354, Taiwan

**Keywords:** acupuncture, chemotherapy, peripheral neuropathy, breast cancer, Semmes–Weinstein monofilament, neuropathic pain

## Abstract

Chemotherapy-induced peripheral neuropathy (CIPN) is a disabling side effect caused by neurotoxic chemotherapy. This randomized controlled trial aimed to evaluate the effect of manual acupuncture on CIPN. Twenty eligible breast cancer patients receiving taxane chemotherapy treatment were recruited and randomly divided into verum acupuncture and sham acupuncture groups. Each group received 15 treatments over 9 weeks. Quantitative tactile detection thresholds were measured using Semmes–Weinstein monofilament testing (SWM). The World Health Organization Quality of Life scale (WHOQOL-BREF), the Functional Assessment of Cancer Therapy/Gynecologic Oncology Group-Neurotoxicity (FACT/GOG-Ntx), and the Brief Pain Inventory-Short Form (BPI-SF) were measured before and after treatment. The between-group comparison of SWM revealed that the verum acupuncture group had more improvement of touch perception thresholds compared to the sham acupuncture group. The average pain severity in the BPI-SF of the verum acupuncture group was significantly lower than that of the sham acupuncture group. There were no significant differences in the FACT/GOG-Ntx trial outcome index and WHOQOL-BREF scores between the acupuncture and sham groups. The results suggest that acupuncture can alleviate the neuropathic pain of CIPN and improve touch perception thresholds.

## 1. Introduction

Chemotherapy-induced peripheral neuropathy (CIPN) is one of the refractory and disabling side effects of neurotoxic chemotherapy among patients with cancer. Chemotherapy-induced peripheral neuropathy (CIPN) is a disabling pain condition [1] involving damage to the structure and function of peripheral motor, sensory, and autonomic neurons [2]. Recent clinical data have reported that the incidence of CIPN has decreased over time. The incidence rate of CIPN is approximately 68.1% after 30 days, with an incidence rate of 60% after 90 days, and 30% of patients maintain CIPN symptoms for more than 6 months [3]. Various chemotherapy drugs, such as platinum, taxanes, vinca alkaloids, epothilones, eribulin, and bortezomib interfere with peripheral neurons and dorsal ganglia during treatment and cause CIPN [4].

Diagnostic methods for CIPN include symptomatic observations of sensory nerve damage, such as needle-like sensations, dullness, numbness, and pain. CIPN is mainly caused by sensory nerve damage compared to motor nerve and autonomic nerve symptoms. Numbness and tingling appear earlier and are more severe than pain [4]. The symptoms are generally symmetrical, initiating in peripheral parts such as the fingers and toes and progressing to the whole body based on the disease severity [5]. The ideal clinical evaluations including clinical examination, objective neurophysiological parameters, and patient-reported outcomes are common diagnostic protocols in CIPN [6]. In addition to nerve conduction studies (NCS), as the gold standard neurophysiological evaluation, quantitative sensory testing (QST) also quantifies the sensory changes in the progress of CIPN. Evaluation methods include vibration threshold detection using a calibrated tuning fork, thermal detection using cold hyperalgesia testing, and sharpness detection through monofilaments. The measurements can be quickly performed to objectively evaluate sensory function [6]. Recently, evaluation of the reliability of monofilaments in neuropathic pain and their interpretation in cancer-related neuropathy has gained the attention of cancer therapists [7,8].

In clinical practice, the interruption of therapy or a change in chemotherapy dose is still the recommended treatment for CIPN; however, this still tends to reduce the effectiveness of chemotherapeutic agents in controlling tumor growth [4]. Currently, the serotonin-norepinephrine reuptake inhibitor, duloxetine, may be recommended as a beneficial clinical treatment for CIPN [4]. However, the clinical use of duloxetine is limited, as it is not universally applicable to all patients and has limited efficacy in persistent CIPN [9]. Acupuncture, a worldwide Chinese medical technique, has demonstrated effectiveness in analgesia and cancer-related symptoms [10]. By analyzing reliable patient-reported outcome measures, several randomized controlled trials revealed preliminary evidence-based treatment effects of acupuncture or electro-acupuncture (EA) in the treatment of CIPN [11,12,13,14]. As to objective assessment tools, although NCS assess both the sensory and motor action potential and provide information about the extent of axonal loss in patients with CIPN [6], NCS parameters may not change until late in the course of chemotherapy [6,15]. Therefore, we conducted a randomized controlled trial using (1) the patient-initiated reporting scale assessment and (2) the quantitative sensory testing, Semmes–Weinstein monofilament (SWM), to evaluate the efficacy of acupuncture on CIPN in patients with breast cancer. 

## 2. Materials and Methods

### 2.1. Trial Design

We conducted this pilot, single-blind, randomized controlled trial at the Tainan Municipal An-Nan Hospital, China Medical University in southern Taiwan. All patients with breast cancer who received neurotoxic chemotherapy were screened for eligibility. Eligible participants were randomly assigned to the acupuncture or sham acupuncture group in a 1:1 ratio. Participants in each group received 15 acupuncture treatments within 9 weeks, including acupuncture treatment twice a week for the first 6 weeks, followed by acupuncture treatment once a week for the next 3 weeks. The research protocol was in compliance with the Declaration of Helsinki and good clinical practice guidelines and was approved by the Research Ethics Committee (CMUH106-REC2-117) of the China Medical University Hospital and registered on Clinicaltrials.gov (NCT03626220). Before randomization, all participants provided signed, written, and informed consent.

### 2.2. Patient Eligibility

The inclusion criteria included adult women aged above 20 years, diagnosed with stage I–III breast cancer by a histological analysis, and who had completed adjuvant or neoadjuvant neurotoxic chemotherapy (including taxane-based or platinum-based), with the severity of CIPN matching the definition of the National Cancer Institute-Common Terminology Criteria for Adverse Events (NCI-CTCAE) 4.0 version more than grade 1. In addition, the patients’ daily physical status matched the definition of the Eastern Cooperative Oncology Group (ECOG) performance status less than grade 3.

Exclusion criteria included an average survival time of fewer than 3 months, a history of diabetic neurological disease before chemotherapy, other pre-existing peripheral neurological diseases, a history of inflammatory or metabolic arthritis, severe coagulopathy or potential bleeding tendency, unstable cardiovascular disease, use of a cardiac pacemaker, or other pre-existing musculoskeletal diseases.

### 2.3. Randomization and Intervention

After screening for inclusion/exclusion criteria, eligible participants signed the written informed consent and completed the baseline assessments that included a quantitative sensory test using the SWM and patient-reported outcome measures. Eligible participants completed the baseline assessments within 7 days before their first acupuncture/sham acupuncture treatment. 

After screening eligibility and completing baseline assessments, participants were randomized to either the verum acupuncture or the sham acupuncture group using a block randomization method [16,17]. The statistician created a randomization sequence using IBM^®^ SPSS^®^ Statistics before study commencement and contained it in sealed envelopes. The balanced sample size was ensured by dividing the participants into blocks of two [16]. All eligible participants and assessors were unaware of the assignment order. Only the physician who practiced the interventions on the participants was aware of the group allocation. Participants were allowed to withdraw from the study at any time. 

According to the study protocol, the physician with a traditional Chinese medicine (TCM) license and with a minimum of 5 years of acupuncture experience performed all treatments. We selected the acupuncture points on the abdomen (Qihai [CV6]), bilateral upper limbs (Quchi [LI11], Neiguan [PC6], and Hegu [LI4]), and bilateral lower limbs (Zusanli [ST36], Sanyingjiao [SP6]). Disposable sterile stainless-steel needles (CASOON, Wuxi Jiajian Medical Instrument Company, LTD, Jiangsu, China), 0.3 mm in diameter and 40 mm length, were inserted into the pre-designated acupoints as shown in Figure S1. In the verum acupuncture group, the treatment depth of the needles was, as per the safe depth of each acupuncture point, about 10–25 mm [18]. After insertion, the TCM practitioner manually twisted the needle to obtain a “de-qi” sensation [19], defined as the practitioner’s feeling of heaviness or tightness from needle manipulation during acupuncture, while the patient felt numbness, soreness, fullness, or heaviness at the needling site. In the sham acupuncture group, sham acupuncture was performed with minimal needling, where the depth of the needle was shallow and less than 4 mm. The location of the needle was approximately 0.5 cun (nearly 1 cm) away from the corresponding acupuncture point. There was no needling operation and the patient did not feel any subjective “de-qi“ sensation. Adverse events were recorded and monitored at each visit [20,21]. From a previous review, the majority of responders receiving acupuncture for the treatment of CIPN reported that their needle retention time was 21–30 min [22]. Therefore, the needle retention time was designed to be 30 min. No needle manipulation was applied in the present study.

### 2.4. Quantitative Sensory Test (QST)

SWM is a clinically easy-to-administer, quantifiable, and reliable tool for evaluating protective sensation loss [23,24]. Measurements were performed using a set of 20 von Frey monofilaments (Semmes–Weinstein von Frey Aesthesiometer, Stoelting Co., Wood Dale, IL, USA), with evaluator size/target forces ranging from 1.65/0.008 g to 6.65/300 g. Each monofilament was calibrated to a target force in grams (g) within a 5% standard deviation [7]. Thresholds for categories of tactile perception were defined by the filament manufacturer as follows: normal (hands/plantar: 0.008–0.07 g/0.008–0.4 g); diminished light touch (hands/plantar: 0.16–0.4 g/0.6–2.0 g); diminished protective sensation (hands/plantar: 0.6–2 g/4–8 g); loss of protective sensation (hands/plantar: 4–180 g/10–180 g); deep pressure sensation only (hands/plantar: 300 g/300 g) [7]. Measurement sites included the sole, tip of the big toe, palm, and tip of the middle finger (Figure S2). All tests were performed by an independent assessor who was blinded to the allocation of randomization. All the procedures followed the operation manual provided by the filament manufacturer.

### 2.5. Patient-Reported Outcome Measures (PROMs)

The Brief Pain Inventory-Short Form (BPI-SF) was measured before the first treatment (baseline value) and at the 3rd, 6th, and 9th weeks after the intervention. The BPI-SF is an instrument widely used to evaluate pain in patients with cancer, including numerative pain severity and pain interference with patients’ daily function [25]. The average pain severity was chosen as the primary assessment of neuropathy symptoms based on its ability to detect neuropathic pain in cancer patients [26]. The BPI-SF assesses pain when responding to the questionnaire (right now) and at its worst, least, and average pain severity in the last 24 h. The items range from 0 to 10 (0 = no pain; 10 = worst pain). The higher the pain severity scores, the worse the degree of pain experienced by the patient. The BPI-SF also measures the pain interference on seven daily functions during the past 24 h, including general activities, mood, walking ability, normal work, relations with other people, sleep, and enjoyment of life. Using numeric scales, the items range from 0 to 10 (0 = no interference; 10= interferes completely) [27]. The higher the pain interference scores, the worse the quality of life. The coefficient alpha for the internal reliability was 0.81 for the severity scale and 0.89 for the interference scale [28]. This study measured the average pain severity and seven pain interference domains for eligible participants.

The Functional Assessment of Cancer Therapy/Gynecologic Oncology Group-Neurotoxicity-13 (FACT/GOG-NTX-13) was measured before the first treatment (baseline value) and at the 3rd, 6th, and 9th weeks after the intervention. It has been evaluated as a reliable and valid scale for the functional assessment of cancer therapy [29,30] and to evaluate the health-related quality of life associated with chemotherapy-induced neurotoxicity [31]. The FACT/GOG-NTX-13 is a 40-item self-report questionnaire that includes the 27-item General Assessment of the Quality of Life scale (FACT-G) alongside its 13-item FACT/GOG-Neurotoxicity (FACT/GOG-Ntx) subscale. The 27-item FACT-G (general version) consists of four domains: physical well-being (PWB), which contains seven items; social well-being (SWB), which contains seven items; emotional well-being (EWB), which contains six items; and functional well-being (FWB), which contains seven items. It evaluates the quality of life of patients undergoing cancer therapy over the past 7 days. The question is rated on a five-point scale, from 0 to 4 (0 = not at all; 4 = very much) and summed (total score range = 0–108). An increase in scores represents an improvement in the overall quality of life. 

The 13-item FACT/GOG-Ntx subscale measures the severity and impact of neurotoxicity symptoms over the past seven days. The instrument assesses sensory symptoms, such as numbness, tingling, and discomfort in hands and feet, motor symptoms such as trouble walking, buttoning buttons, and ototoxicity, such as ringing or buzzing in the ear [32]. Items are rated on a five-point scale, scored from 0 to 4 (0 = not at all; 4 = very much) and summed (total score range = 0–52). Higher scores on the neurotoxicity scale indicate improvements in neurotoxic symptoms. 

The 40-item FACT/GOG-Ntx total score represents the sum of the FACT-G and FACT/GOG-Ntx subscale (total score range = 0–160). The FACT/GOG-Ntx trial outcome index (TOI) contained the scores of the PWB, FWB, and FACT/GOG-Ntx subscale (total score range = 0–104). The TOI assesses changes in neurological function and quality of life in terms of physical fitness and functional recovery without including the assessment of participants’ emotional and social ability recovery. The two scores were significantly different in patients who received chemotherapy and changed sensitively over time [31].

The WHOQOL-BREF Taiwan version was validated as a good clinical tool across five kinds of Taiwanese cancer survivors, including head/neck, colorectal, liver, lung, and gynecologic cancers [33]. It was developed using standard translation procedures and psychometric evaluations, which include four areas: physical health, psychological state, social relationships, and environmental adaptation. There was a total of 28 items by adding two additional items to account for Taiwanese cultural adaptations. In addition to two area-based items, the remaining 26 items were distributed into four domains: the physical health domain includes seven items; second, the psychological domain includes six items; third, the social relationships domain includes four items; and fourth, the environment domain includes nine items. Item scores range from 1 to 5 (1 = the worst condition; 5 = the best condition), except for three items (Ph1, Ph2, and Ps6), which are reverse-coded. Each domain in the scale can be translated into two ranges of domain scores (0–100 and 4–20), and we used a 0–100 scale in this study. Higher scores in each domain indicate a better quality of life for patients with cancer [33].

### 2.6. Statistical Analysis

All data were analyzed using IBM^®^ SPSS^®^ Statistics version 22 (Statistical Product and Service Solutions Statistics, I.B.M., Inc., Armonk, NY, USA). For demographic data, continuous data were analyzed using a two-sample *t*-test or Mann–Whitney *U* test, while categorical data were analyzed using Fisher’s exact test. If the data of each group failed to pass the Shapiro–Wilk normal distribution test (all *p* value < 0.05), the between-group differences were examined using the nonparametric Mann–Whitney *U* test, and the intra-group differences were examined using the Wilcoxon signed-rank test. If the measurements of the baseline values were as per the Shapiro–Wilk normal distribution test (all *p* value > 0.05), the difference between groups was examined using a two-sample *t*-test, and the intra-group difference was examined using a paired *t*-test. If the study groups fit the normal distribution, the two-way ANOVA with repeated measures was used to evaluate the interaction of group and time factors. Differences between or within groups were considered significant at *p* < 0.05. 

## 3. Results

### 3.1. Demographics

**Figure 1:**
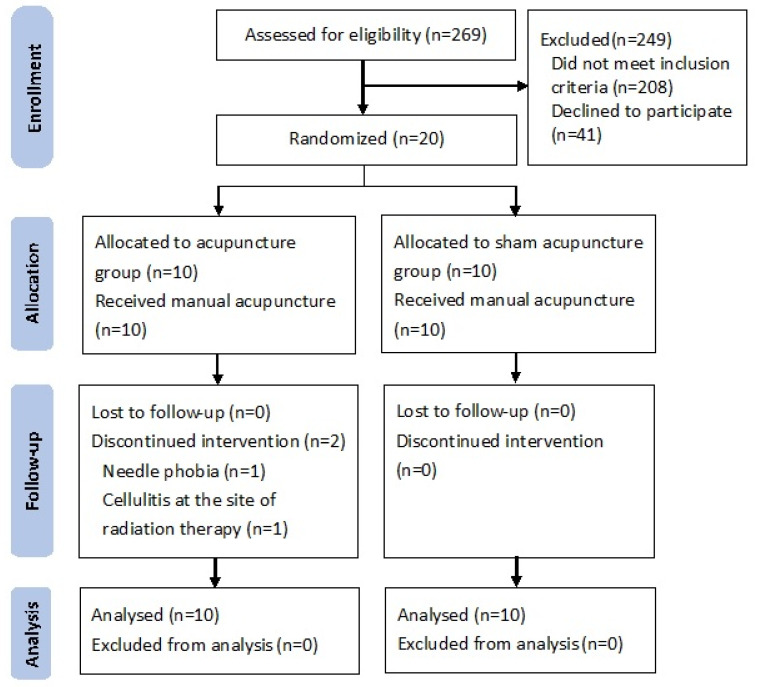
CONSORT flow chart.

In this study, 269 patients diagnosed with breast cancer were screened during the study period from June 2018 to July 2020 (Figure 1). Among 269 patients, 208 did not meet the inclusion criteria, while 61 patients were eligible. Among the 61 eligible patients, 41 refused to participate in the study, and 20 were randomly assigned to the verum acupuncture and sham acupuncture groups. Among the 20 selected participants, none dropped out before treatment, while two dropped out during treatment (10%), and a total of 18 (90%) completed the interventional procedure. Of the two participants who dropped out of the study intervention, one had needle phobia and only completed three acupuncture interventions, while the other patient had cellulitis due to radiotherapy and completed six acupuncture interventions. The results of 20 participants were included in the statistical analysis. 

The basic sociodemographic data of the eligible participants are listed in Table 1. Among the 20 participants, the distribution of breast cancer stages included 20%, 35%, and 45% for the first, second, and third stages, respectively. All participants received docetaxel treatment, and 15% of them also received carboplatin treatment. The CIPN classification status assessed using the NCI-CTCAE is the first level, accounting for 85%, and the second level, accounting for 15%. In all basic sociodemographic data, there was no difference between the two groups, including age, sex, body mass index (BMI), history, family history, occupation, breast cancer stage, chemotherapy type, cumulative dose, ECOG stage, NCI-CTCAE classification, and the average follow-up days from the end of chemotherapy to the acupuncture treatment.

### 3.2. Primary Outcome

#### Effects of Acupuncture on Average Pain Severity

**Figure 2:**
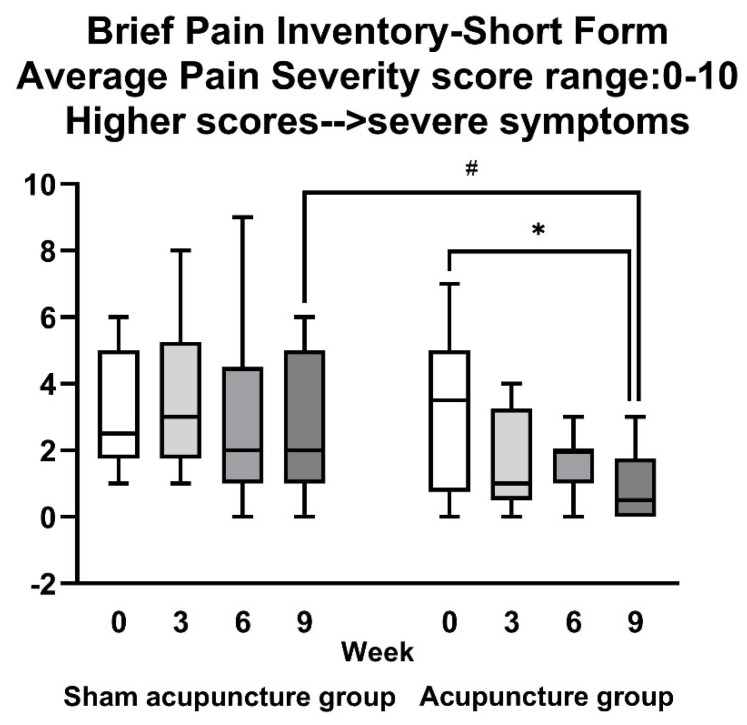
Average pain severity scores in BPI-SF during the treatment periods. Data are expressed as mean ± standard error of the mean (SEM). * *p* < 0.05 represents the within-group values analyzed using the Wilcoxon signed-rank test. # *p* < 0.05 represents the between-groups values at the 9th week analyzed using the Mann–Whitney *U* test. Abbreviations: BPI-SF, Brief Pain Inventory-Short Form.

A BPI-SF measurement was performed before (baseline value), and in the 3rd, 6th, and 9th weeks after acupuncture or sham intervention. The scale included the evaluation of pain severity and pain interference. We chose the average pain severity value and analyzed pain interference, including seven domains. The results are shown in Figure 2 and Table S1. The group data collected in BPI-SF in Figure 2 did not fit the normal distribution. Therefore, the non-parametric analysis was adopted; a between-group comparison was tested by the Mann–Whitney *U* test while the within-group comparison was tested by the Wilcoxon signed-rank test [34]. There was no significant difference between the two groups in the baseline of the average pain severity value (verum acupuncture group: 3.10 ± 2.33 vs. sham acupuncture group: 3.10 ± 1.79; *p* = 0.974). The intra-group comparison of the verum acupuncture group revealed that the average pain severity significantly reduced at the 9th week (*p* = 0.031) compared with the baseline. The intra-group comparison of the sham acupuncture group showed no significant difference in the 3rd, 6th, and 9th weeks after treatment. The between-group comparison showed that the average pain severity of the verum acupuncture group at the 9th week was significantly lower compared to the sham acupuncture group (verum acupuncture group: 0.88 ± 1.13 vs. sham acupuncture group: 2.70 ± 2.00; *p* = 0.039).

### 3.3. Secondary Outcomes

#### 3.3.1. Effects of Acupuncture on Pain Interference

The results of the seven-dimensional analysis of the pain interference values are presented in Figure S3 and Table S1. There was no significant difference between the two groups in the baseline values of each aspect except the pain interfering with the walking ability. The intra-group comparison revealed that there was a significant intra-group difference in the sham acupuncture group at the 3rd week regarding the degree of pain that interfered with normal work. The between-group comparison revealed that, regarding the degree of pain interfering with sleep, the acupuncture group had a significantly lower degree of pain interfering with general work and sleep at the 3rd week than the sham acupuncture group. However, there was no other significant difference found between the two groups. 

#### 3.3.2. Effects of Acupuncture on Quantitative Sensory Test

**Figure 3:**
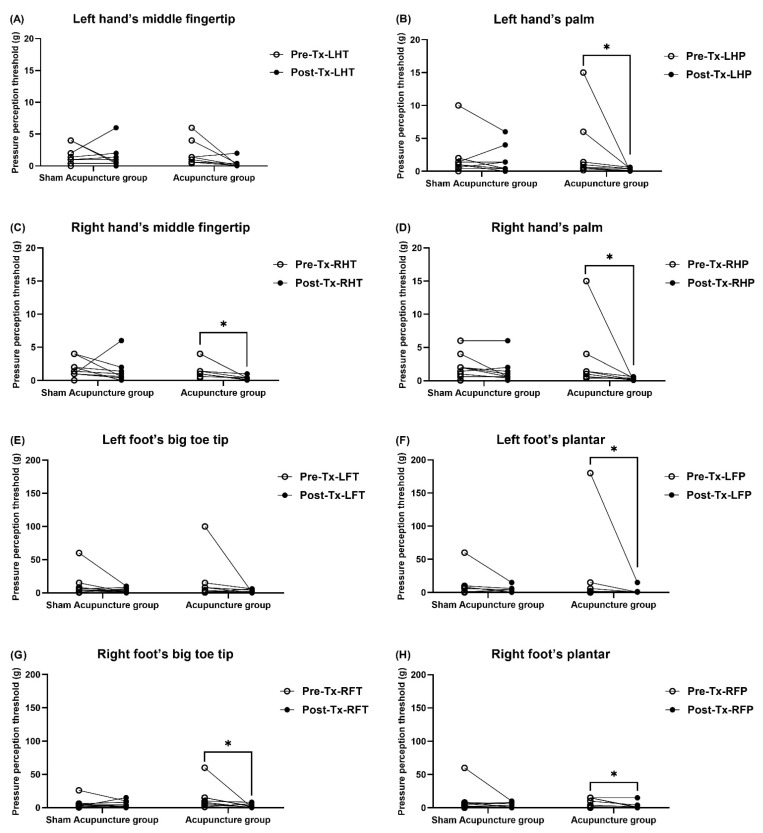
(**A**–**H**) Mechanical touch-pressure detection thresholds at the fingers, palms, toes, and plantars using Semmes–Weinstein monofilament evaluation measured before and at the 9th week after 15 treatment sessions. * *p* < 0.05 represents the within-group values validated using the Wilcoxon signed-rank test. Abbreviations: LHT, Left hand’s middle fingertip; LHP, Left hand’s palm; RHT, Right hand’s middle fingertip; RHP, Right hand’s palm; LFT, Left foot’s big toe tip; LFP, Left foot’s plantar; RFT, Right foot’s big toe tip; RFP, Right foot’s plantar.

Von Frey monofilament fibers were used to test the touch perception thresholds before and after the 9-week treatment. The inspection points included eight locations: the left hand’s middle fingertip (LHT), left hand’s palm (LHP), right hand’s middle fingertip (RHT), right hand’s palm (RHP), left foot’s big toe tip (LFT), left foot’s plantar (LFP), right foot’s big toe tip (RFT), and right foot’s plantar (RFP) (Figure S2). The results illustrated in Figure 2 and Figure 3 show that the baseline touch perception thresholds of the two groups were higher than the normal range. According to the von Frey monofilament fiber manufacturer, the normal range of touch perception threshold is that the hand threshold is lower than 0.07 g and the foot threshold is lower than 0.4 g [7]. There was no significant difference between the two groups before treatment. After the 9-week treatment, the intra-group comparison of the verum acupuncture group showed that the touch perception thresholds of LHP, RHT, RHP, LFP, RFT, and RFP detection points were significantly improved, except for the LHT and LFT (Figure 3B–D,F–H). The intra-group comparison of the sham acupuncture group showed no significant difference between all the detection points before and after acupuncture (Table S2).

**Figure 4:**
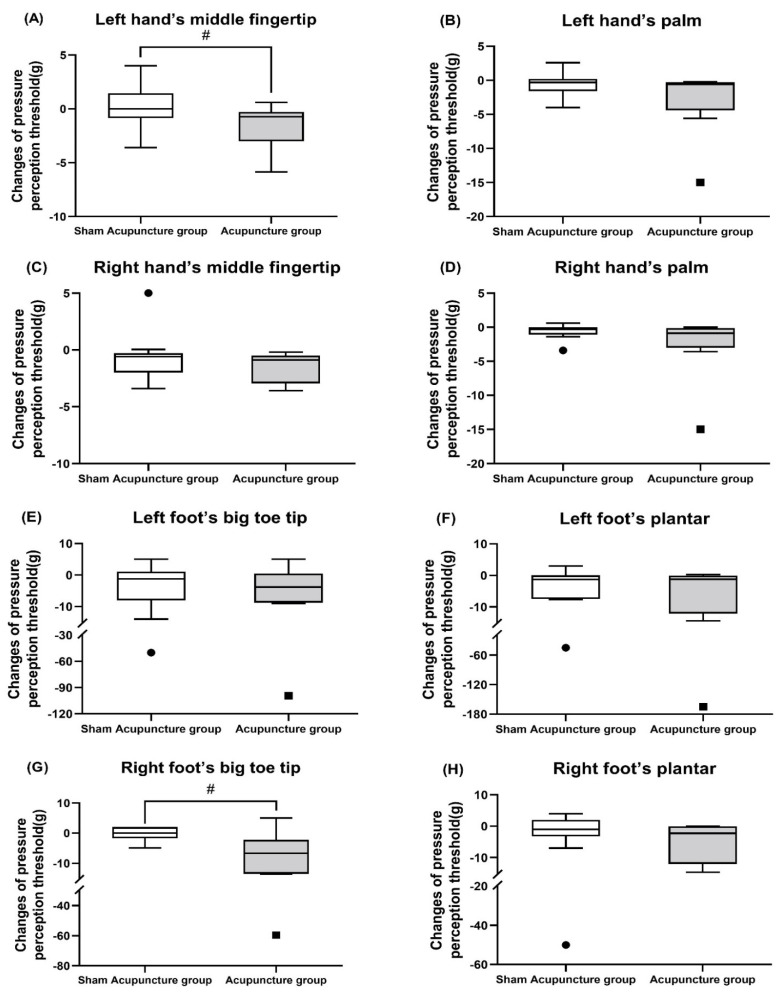
(**A**–**H**) Changes in the mechanical touch-pressure perception before and after the 15 treatment sessions threshold. Data are presented as minimum and maximum values and lower, middle, and upper quartiles for each group. # *p* < 0.05 represents the between-group values validated by the Mann–Whitney *U* test.

Regarding the between-group comparison (verum acupuncture and sham acupuncture groups), the change in the touch perception threshold before and after treatment was significantly different in the LHT (acupuncture group: −1.54 ± 2.12 vs. sham acupuncture group: 0.20 ± 2.51; *p* = 0.049) and the RFT (acupuncture group: −12.45 ± 19.99 vs. sham acupuncture group: −0.34 ± 7.15; *p* = 0.041) (Figure 4A,G and Table S2).

#### 3.3.3. Effects of Acupuncture on Chemotherapy-Induced Neurotoxicity

**Figure 5:**
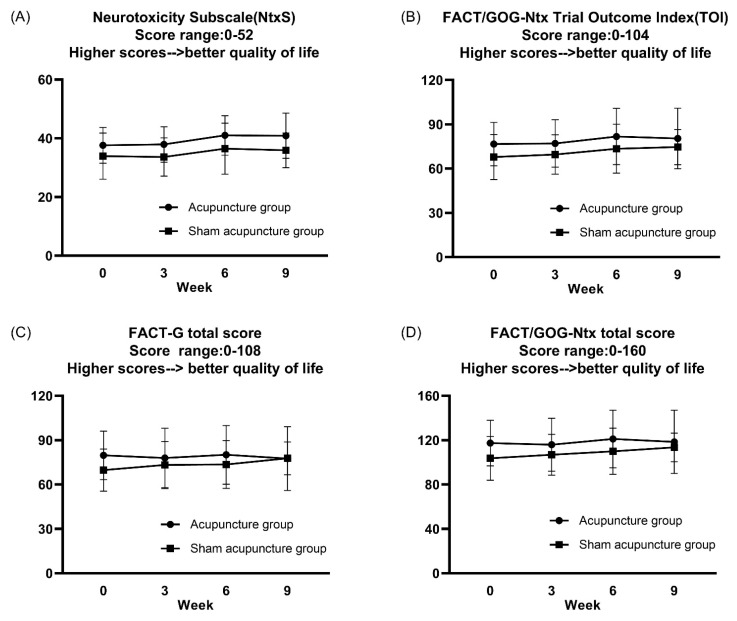
The participants’ FACT/GOG-Ntx scale before (baseline value), and at the 3rd, 6th, and 9th weeks. (**A**) Patient-reported outcomes in the neurotoxicity subscale, which evaluates the degree of neurotoxicity. (**B**) Patient-reported outcomes in the FACT/GOG-Ntx trial outcome index (TOI), which contains the neurotoxicity subscale, physical and functional well-being in quality of life. (**C**) Patient-reported outcomes in the FACT/GOG-G total score, which contains physical, social/family, emotional, and functional well-being in quality of life. (**D**) Patient-reported outcomes in the FACT/GOG-Ntx total score. Data are expressed as mean ± standard error of the mean (SEM). The two-way ANOVA with repeated measures revealed that there were neither significant interaction effects between time and group factors nor significant main effects of time and group factors.

The participants’ FACT/GOG-Ntx scale was tested before (baseline value), and at the 3rd, 6th, and 9th weeks after acupuncture or sham intervention. The results are shown in Figure 5. The two-way ANOVA with repeated measures revealed that there were neither significant interaction effects between time and group factors nor significant main effects of time and group factors (Figure 5).

#### 3.3.4. Effects of Acupuncture on WHOQOL-BREF

The World Health Organization Quality of Life scale (WHOQOL-BREF) was measured before (baseline value) and at the 9th week of treatment, and the results are shown in Table S3. The results revealed that the intra-group comparison in the sham-controlled group on the psychological domain was significantly improved after treatment. However, there were no significant improvements in physical health, social relationships, and environmental domains.

### 3.4. Adverse Events

No patients experienced adverse effects, such as all-cause mortality, serious adverse events, or other adverse events in our study. 

## 4. Discussion

To the best of our knowledge, this study is the first randomized, sham-controlled clinical trial to evaluate the effect of acupuncture for CIPN combined with the use of SWM as an objective quantitative sensory assessor and patient-initiated reporting scale assessment. 

According to the American Society of Clinical Oncology (ASCO) guideline for management of chemotherapy-induced peripheral neuropathy, duloxetine is the only recommendation on the treatment of CIPN [4,35]. In the randomized controlled trial investigating the effects of duloxetine, the average pain severity in BPI-SF was adopted as the primary endpoint [26]. In the present study, we set the average pain severity in BPI-SF as the primary endpoint. The results of the present study indicate that acupuncture can alleviate neuropathic pain in patients with cancer, and the average pain severity in BPI-SF at the 9th week after the intervention was significantly more improved in the verum acupuncture group than the sham acupuncture group. In the pre- and post-treatment comparison of the acupuncture group, the mean pain severity was significantly lower at the 9th week compared to the baseline values. Several randomized controlled studies have reported similar results, as detailed below. In a recent pilot randomized clinical trial, the true acupuncture group (*n* = 24) received a set of auricular and EA treatments, while the sham treatment group (*n* = 23) received non-acupuncture, non-insertion procedures, and a usual care group (*n* = 21). By assessing numerical rating scales, the EA group had a significantly better mean absolute reduction in neuropathic pain than the sham group, with the least reduction in the usual care group [14]. In another pilot randomized controlled trial, the mean pain severity at week 8 in the EA group (*n* = 20) was lower than that in the usual care group (*n* = 18) [13]. Molassiotis et al. showed that the worst severe pain in the BPI-SF at week 8 was significantly improved in the manual acupuncture group (*n* = 44) compared to the usual care group (*n* = 43) [12]. However, these two studies did not include a sham control group design. The present study adopted a sham control group design with the blindness of patients [36]. The results of our study also confirmed that manual acupuncture treatment reduced pain in patients with CIPN.

SWM is a QST tool and is the only manual test used to examine sensory perception thresholds [37]. SWM is a highly reliable and sensitive measure when applied to appropriate psychophysical modalities to assess tactile point pressure sensitivity [37]. SWM is unlike the traditional standardized assessment of somatosensory function that examines grossly myelinated peripheral nerve fibers, including the corresponding central pathways [38]. It has been suggested that assessing appropriate sensory performance patterns through QST may help to identify certain treatments responsive to underlying pain mechanisms [38]. On application to the assessment of CIPN, tactile detection thresholds using 20 von Frey filaments were shown to be negatively correlated with the FACT-GOG-Ntx upper and lower extremity numbness scores, while being positively correlated with the Neuropathic Pain Scale (which uses a 0–10 numerical rating scale to quantify pain intensity across the territory) [7]. 

Few randomized controlled trials of acupuncture for CIPN have used SWM as an assessment tool to evaluate tactile perceptual sensory recovery [39,40]. In our study, regarding the change in post-treatment touch sensory detection thresholds before and after treatment, the verum acupuncture group showed significant improvements in the left hand’s middle fingertip and right foot’s big toe tip compared to the sham acupuncture group (*p* < 0.05). In addition, in the pre- and post-treatment comparison, participants in the acupuncture group showed significant improvements in touch pressure perception thresholds at six of eight test points after 15 acupuncture sessions over 9 weeks compared to the pre-treatment period (*p* < 0.05). 

As found in Hsieh’s study, 12 laser acupuncture sessions alleviated the effects of oxaliplatin on peripheral neuropathy in patients with advanced gastrointestinal cancer in a single-arm observational study design. Laser acupuncture significantly relieved oxaliplatin-induced cold trigger pain (using the cold-water immersion test) and anomalous mechanical pain (using von Frey). However, this study did not include a sham control group to compare with laser acupuncture [39]. In Wong’s prospective phase II study, changes in hand and foot numbness scores were assessed using three von Frey filaments pre-designated as one to three points each [40]. Patients treated using neurotoxic chemotherapy with 12 sessions of transcutaneous nerve stimulation showed a significant reduction in numbness scores in the palms, fingers, feet, and toes at 6 months from the baseline value. However, the study did not investigate whether EA affects the numbness scores by von Frey filaments. The aforementioned studies used SWM as an assessment tool to assess touch sensory recovery; however, there is a lack of randomized controlled studies designed to assess the efficacy of acupuncture on CIPN using SWM as an objective assessment tool.

Four strategies have been proposed for designing control groups used alone or in combination with acupuncture RCTs: (1) the absence of acupuncture needle insertion, (2) a different location of inserted acupuncture needles, (3) a different depth of insertion, and (4) the use of assistant tools [36]. However, a recent study reported that real acupuncture needling exhibits a greater insertion force and pullout force compared with a non-penetrating sham needle and that the participants were able to discriminate between real acupuncture and sham acupuncture [41]. Therefore, the control group design of the present study did not use the non-penetrating control while adopted the strategies of a different location of inserted acupuncture needles and a different depth of insertion.

In regard to different locations for inserted acupuncture needles, sham acupoints comprised the points drifting off the specific acupoints about 0.5 B-cun (about 1 cm). In regard to the different depth of insertion, the sham acupuncture adopted the depth of less than 4 mm, which was considered not causing “de-qi” [36].

According to the review by Lu et al. [22], the most frequently used acupoints for the treatment of CIPN were located in the hands and feet for the treatment of CIPN, such as Taichong (LR3), and Hegu (LI4), Ba Feng, Ba Xie, Zusanli (ST36), Sanyinjiao (SP6), Quchi (LI11), Waiguan (TB5), Yanglingquan (GB34), Taixi (KI3), Xuehai (SP10), Jiexi (ST41), and Lieque (LU7) [22]. A previous study reporting laser acupuncture attenuated oxaliplatin-induced peripheral neuropathy in patients with gastrointestinal cancer used acupoints Neiguan (PC6), Daling (PC7), Laogong (PC8), Zhongchong (PC9), and Shaoshang (LU11) in the interior side of upper extremities, and Sanyinjioa (SP6), KI3 (Taixi), Kunlun (BL60), Yongquan (KI11), and Rangu (KI12) in the lower extremities [39]. Most of the acupoints were selected in the interior side of four limbs. In addition to the points mentioned above, Qihai (CV6) on the abdomen was also commonly used for CIPN [42]. Based on these studies [22,39,42], we selected the acupoints on the abdomen (Qihai [CV6]), bilateral upper limbs (Quchi [LI11], Neiguan [PC6], and Hegu [LI4]), and bilateral lower limbs (Zusanli [ST36], Sanyingjiao [SP6]). In the theory of traditional Chinese medicine, these acupoints are for tonifying Qi, regulating Qi and blood circulation, and treating localized symptoms. It is noted that a recent study found that acupuncture relieved CIPN in breast cancer patients using similar acupoints [13].

It is noteworthy that acupuncture has been widely recommended in cancer care and cancer-related symptoms, including cancer pain and neuropathy [10]. Acupuncture may exert its analgesic effect through the gate-control mechanism in the spinal cord [43,44,45]. However, the mechanism of the analgesic effect related to chemotherapy-related neuropathic pain remains unclear. Some studies have demonstrated that repeated EA stimulation attenuates paclitaxel-induced neuropathic pain through the mediation of the spinal opioid receptor, α2 and β-adrenoceptors [46]. Furthermore, the mechanism by which acupuncture improves motor and sensory nerve function remains unknown. This may not be explained by increasing endorphin levels [47]. Recent animal studies have shown that acupuncture and EA accelerate nerve regeneration in a silicon rubber tube tubulated study [48,49], and are even beneficial to neuronal stem cell regeneration with a significant increase in the mRNA expression levels of multiple neuronal genes in the skin [50]. Further studies are needed to investigate the mechanisms of action of acupuncture on the neurological changes in CIPN.

Previous randomized controlled clinical trials using FACT-NTX as the assessment modality revealed that EA or manual acupuncture improved the quality of life of patients with CIPN after neurological impairment [12,13]. In our study, there was no significant difference between the verum acupuncture group and sham acupuncture group. However, given the small sample size of the present study, a large clinical trial should be conducted in the future for further evaluation.

As our study aimed to explore the efficacy of manual acupuncture on CIPN by using SWM as a quantitative sensory test, there are still limitations in the study that need to be pointed out. First, this was a pilot randomized controlled trial, and the small sample size limited the effectiveness of acupuncture, and a larger randomized controlled trial should be considered in the future. Second, the eligible participants in our study were limited to patients with type I–III breast cancer and most received docetaxel-based chemotherapy. Future studies may consider investigating the study design of acupuncture for other cancer types or expanding neurotoxic chemotherapy types. Third, a long-term follow-up is recommended to determine the effect of acupuncture on CIPN. Finally, the current study did not specify the duration of CIPN symptoms as an inclusion criterion. The present study recruited patients with an average CIPN duration of approximately 6 months. However, considering the trend that the incidence of CIPN decreases over time, the duration of CIPN should be considered in future studies.

## 5. Conclusions

It is concluded that manual acupuncture alleviated neuropathic pain in CIPN. In addition, manual acupuncture was effective in improving touch perception thresholds, as assessed by SWM. The current results did not reveal that acupuncture improves the quality of life of patients with cancer. This warrants further large-scale clinical trials in the future.

## Figures and Tables

**Table 1:** Baseline sociodemographic data of eligible participants.

Baseline Sociodemographic Data	Acupuncture Group (*N* = 10)	Sham Acupuncture Group (*N* = 10)	Total (*N* = 20)	*p*-Value
Age (years)	47.10 (11.07)	52.10 (11.18)	49.60 (11.13)	0.33 ^§^
Sex (Female/Male)	10/0	10/0	20/0	-- ^‡^
BMI	24.96 (5.99)	23.34 (2.79)	24.15 (4.62)	0.45 ^§^
Past history ^†^				
DM	0 (0)	0 (0)	0 (0)	-- ^‡^
HTN	1 (10)	1 (10)	2 (10)	1.00 ^‡^
Others	0 (0)	2 (20)	2 (10)	0.47 ^‡^
None	7 (70)	5 (50)	12 (60)	0.65 ^‡^
Family history ^†^				
Breast cancer	1 (10)	1 (10)	2 (10)	1.00 ^‡^
Other cancer	5 (50)	6 (60)	11 (55)	1.00 ^‡^
Other diseases	3 (30)	2 (20)	5 (25)	1.00 ^‡^
None	3 (30)	1 (10)	4 (20)	0.58 ^‡^
Occupation				1.00 ^‡^
Employed	5 (50)	4 (50)	9 (45)	
Unemployed	5 (50)	6 (60)	11 (20)	
Breast cancer stages				0.52 ^‡^
I	1 (10)	3 (30)	4 (20)	
II	3 (30)	4 (40)	7 (35)	
III	6 (60)	3 (30)	9 (45)	
Type of Chemotherapy ^†^				
Docetaxel	10 (100)	10 (100)	20 (100)	-- ^‡^
Carboplatin	1 (10)	2 (20)	3 (15)	1.00 ^‡^
Other taxanes	0 (0)	0 (0)	0 (0)	-- ^‡^
Other platinum	0 (0)	0 (0)	0 (0)	-- ^‡^
Cumulative doses (mg)				
Docetaxel	635.80 (119.82)	622.80 (78.18)	629.30 (98.70)	0.78 ^§^
Carboplatin	474 (1498.92)	864 (1835.47)	669.00 (1643.20)	0.74 ^¶^
ECOG				1.00 ^‡^
0	10 (100)	9 (90)	19 (95)	
1	0 (0)	1 (10)	1 (5)	
NCI-CTCAE				1.00 ^‡^
1	8 (80)	9 (90)	17 (85)	
2	2 (20)	1 (10)	3 (15)	
Interval from the end of chemotherapy to acupuncture treatment (days)	277.90 (464.01)	115.90 (101.98)	196.90 (337.37)	0.64 ^§^

Note: Data are presented as mean (standard deviation) or number (percentage). Abbreviations: SD, standard deviation; BMI, body mass index; DM, diabetes mellitus; HTN, hypertension; ECOG, Eastern Cooperative Oncology Group; NCI-CTCAE, National Cancer Institute-Common Terminology Criteria for Adverse Events. ^†^ Reflects the number and percentage of participants answering “yes” to this question. ^‡^ Data are validated using Fischer’s exact test. ^§^ Data are validated by a two-sample *t*-test. ^¶^ Data are validated using the Mann–Whitney *U* test. -- Unable to calculate as there are either too few or no events. No significant difference was observed between groups.

## Data Availability

The data presented, the datasets used and analyzed during the current study are available from the corresponding author on reasonable request.

## Supplementary Materials

The following are available online at https://www.mdpi.com/article/10.3390/jcm10163694/s1, Figure S1. Acupuncture point locations: (A) Quchi(LI11), Hegu (LI4), (B) Neiguan (PC6), (C) Qihai (CV6), Zusanli (ST36), and Sanyingjiao (SP6). Figure S2. Locations where quantitative sensory testing is performed using the Semmes-Weinstein monofilament examination. Figure S3. (A–G) reveal pain interference scores in the BPI-SF during the treatment period. Data are expressed as mean± standard error of the mean (SEM). Table S1. Results of patient-reported outcome in the Brief Pain Inventory-Short Form. Table S2. Results of mechanical touch-pressure perception thresholds at fingers and toes. Table S3. Results of patient-reported outcome in the WHOQOL-BREF (Taiwan version).
