# Supplementary material for: Acupuncture Relieved Chemotherapy-Induced Peripheral Neuropathy in Patients with Breast Cancer: A Pilot Randomized Sham-Controlled Trial
Source: J Clin Med. 2021 Aug 20;10(16):3694. doi: 10.3390/jcm10163694 (PMC8397157; doi:10.3390/jcm10163694)
Supplement: Supplementary file 1 [file jcm-10-03694-s001.zip › jcm-1262968-supplementary.pdf]

Supplementary materials

For

Acupuncture Relieved Chemotherapy-Induced Peripheral Neuropathy in Patients with Breast Cancer:A Pilot Randomized Sham-controlled Trial

Chien-Chen Huang, Tsung-Jung Ho, Hsin-Yueh Ho, Pei-Yu Chen, Cheng-Hao Tu, Yu-Chuen Huang, Yu-Chen Lee, Mao-Feng Sun, Yi-Hung Chen

Figure S1

Figure S2

Figure S3

Table S1

Table S2

Table S3

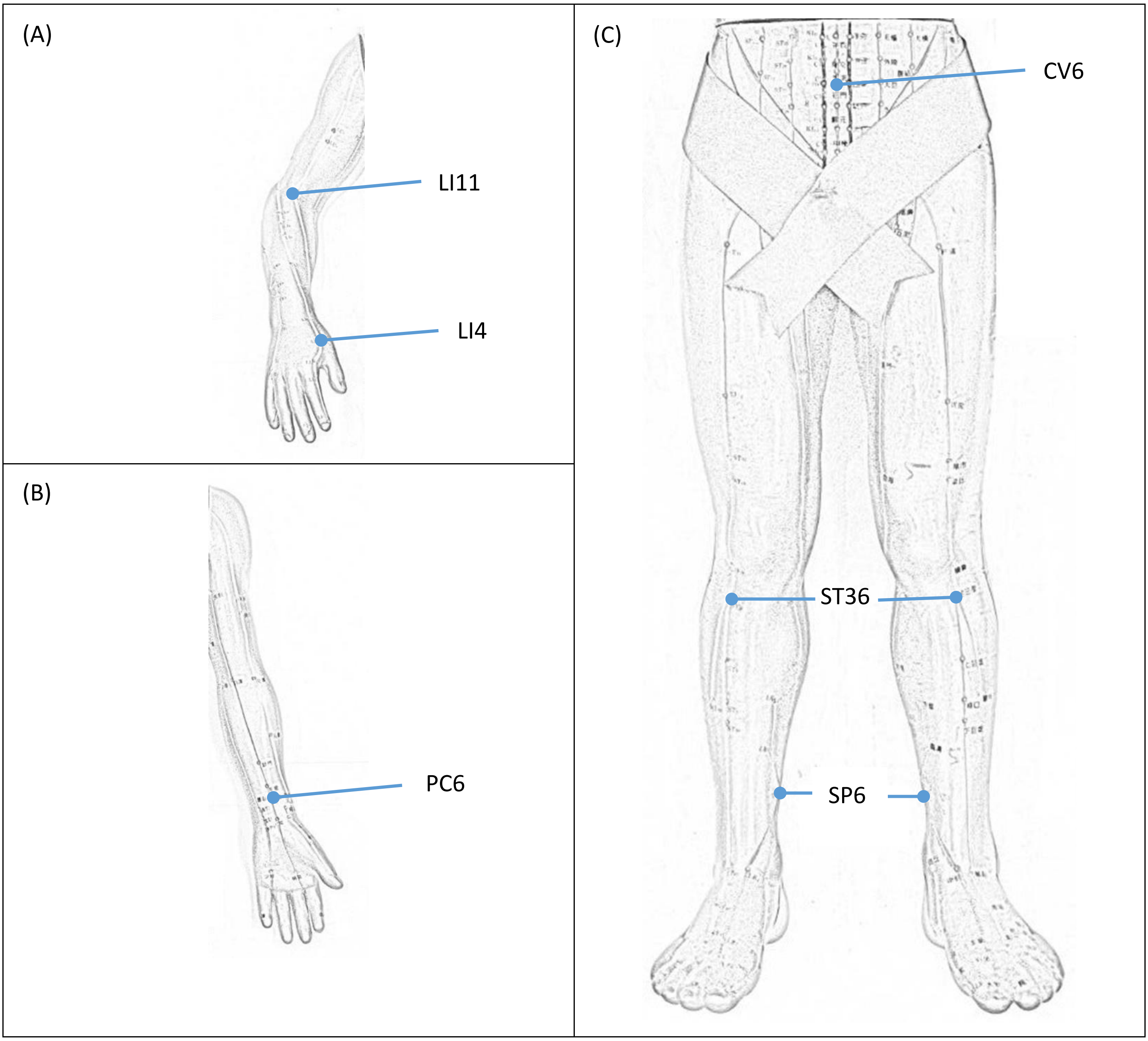

Figure S1. Acupuncture point locations: (A) Quchi(LI11), Hegu (LI4), (B) Neiguan (PC6), (C) Qihai (CV6), Zusanli (ST36), and Sanyingjiao (SP6).

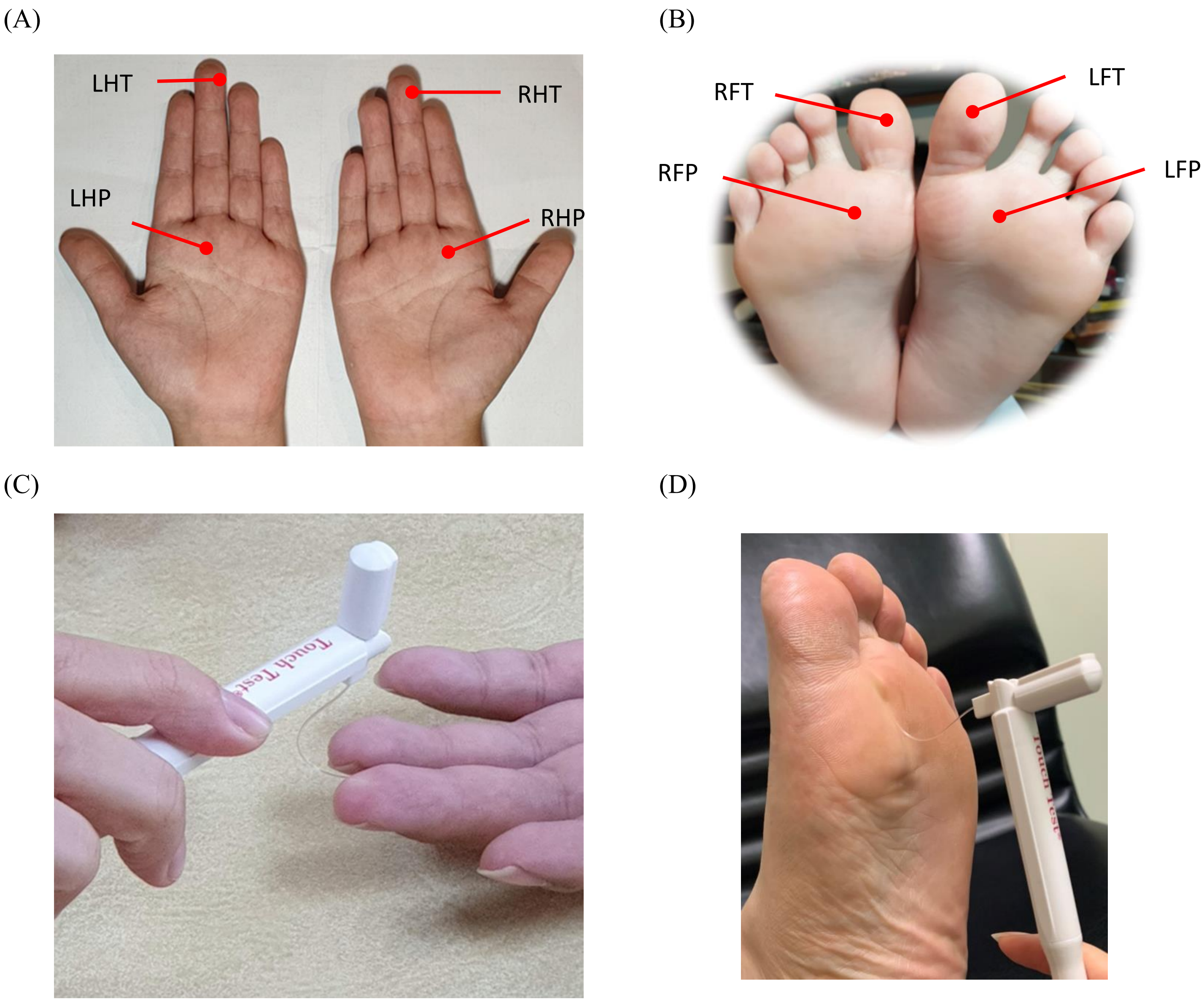

Figure S2. Locations where quantitative sensory testing is performed using the Semmes-Weinstein monofilament examination. (A) Middle fingertips and palms (B) Big toe tips and planters. (C) The touch-press method at hand: press the filament at a 90° angle against the skin until it bows. Hold in place for 1.5 s and then remove (D) The same touch-press method is performed at the foot. Abbreviations: LHT, Left hand's middle fingertip; LHP, Left hand's palm; RHT, Right hand's middle fingertip; RHP, Right hand's palm; LFT, Left foot's big toe tip; LFP, Left foot's plantar; RFT, Right foot's big toe tip; RFP, Right foot's plantar.

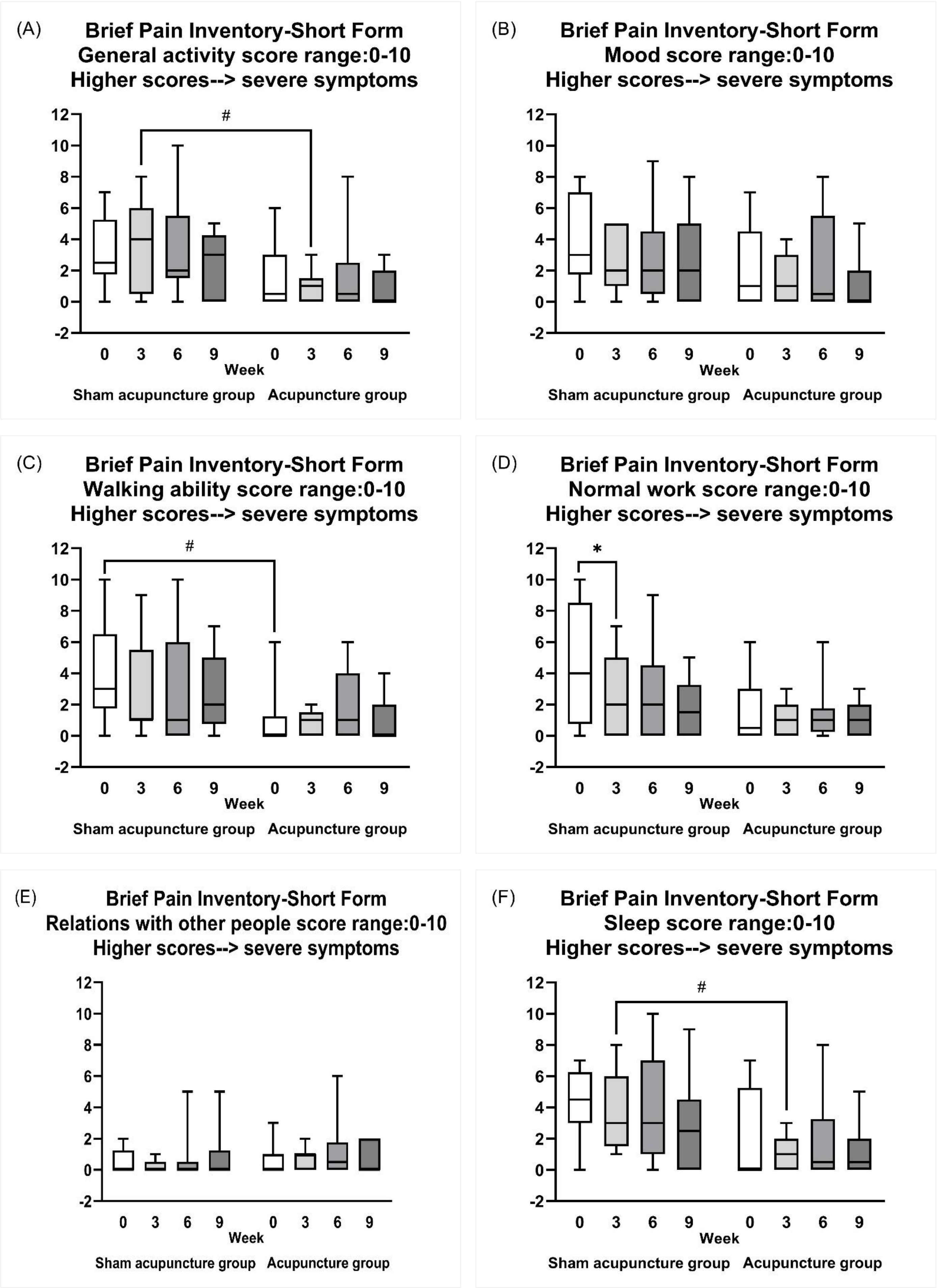
(A)
Brief Pain Inventory-Short Form
General activity score range:0-10
Higher scores--> severe symptoms
#
12
10
8
6
4
2
0
-2
0 3 6 9 0 3 6 9
Week
Sham acupuncture group Acupuncture group
(B)
Brief Pain Inventory-Short Form
Mood score range:0-10
Higher scores--> severe symptoms
12
10
8
6
4
2
0
-2
0 3 6 9 0 3 6 9
Week
Sham acupuncture group Acupuncture group
(C)
Brief Pain Inventory-Short Form
Walking ability score range:0-10
Higher scores--> severe symptoms
#
12
10
8
6
4
2
0
-2
0 3 6 9 0 3 6 9
Week
Sham acupuncture group Acupuncture group
(D)
Brief Pain Inventory-Short Form
Normal work score range:0-10
Higher scores--> severe symptoms
*
12
10
8
6
4
2
0
-2
0 3 6 9 0 3 6 9
Week
Sham acupuncture group Acupuncture group
(E)
Brief Pain Inventory-Short Form
Relations with other people score range:0-10
Higher scores--> severe symptoms
12
10
8
6
4
2
0
-2
0 3 6 9 0 3 6 9
Week
Sham acupuncture group Acupuncture group
(F)
Brief Pain Inventory-Short Form
Sleep score range:0-10
Higher scores--> severe symptoms
#
12
10
8
6
4
2
0
-2
0 3 6 9 0 3 6 9
Week
Sham acupuncture group Acupuncture group

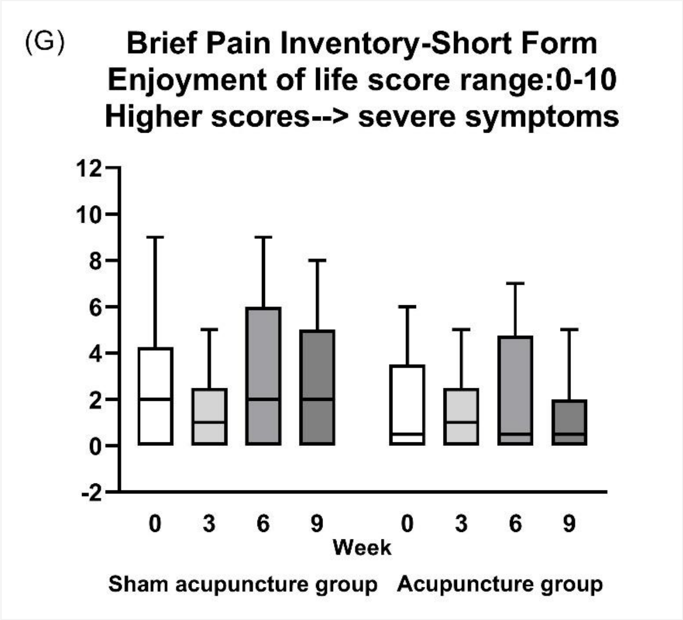

Figure S3. (A) to (G) reveal pain interference scores in the BPI-SF during the treatment period. Data are expressed as mean± standard error of the mean (SEM). *p<0.05, Wilcoxon signed-rank test #p<0.05, after applying the Mann-Whitney *U* test. Abbreviations: BPI-SF, brief pain inventory short form.

Table S1. Results of patient-reported outcome in the Brief Pain Inventory-Short Form

Brief Pain Inventory-Short Form	Acupuncture group			Sham acupuncture group			Mann-Whitney *U* test *p*-value
	N	Mean(SD)	Wilcoxon signed ranks test *p*-Value	N	Mean(SD)	Wilcoxon signed ranks test *p*-Value	
**Average pain severity**							
Baseline	10	3.10(2.33)		10	3.10(1.79)		0.974
Week 3	9	1.72(1.48)	0.172	10	3.50(2.27)	0.781	0.078
Week 6	8	1.63(0.92)	0.063	9	2.78(2.82)	1.000	0.679
Week 9	8	0.88(1.13)	0.031*	10	2.70(2.00)	0.508	0.039*
Changes at week 6	8	-1.63(1.85)		9	-0.11(2.71)		0.288
Changes at week 9	8	-2.38(2.39)		10	-0.40(2.07)		0.078
**General activity**							
Baseline	10	1.40(2.01)		10	3.30(2.31)		0.059
Week 3	9	0.89(1.05)	0.688	9	3.56(2.88)	0.766	0.044*
Week 6	8	1.63(2.77)	1.000	9	3.33(3.12)	1.000	0.105
Week 9	8	0.88(1.25)	0.625	10	2.60(2.01)	0.465	0.063
Changes at week 6	8	0.00(3.02)		9	0.22(1.86)		0.881
Changes at week 9	8	-0.75(2.31)		10	-0.70(2.11)		0.384
**Mood**							
Baseline	10	2.20(2.66)		10	3.60(2.76)		0.190

Week 3	9	1.33(1.66)	1.000	9	2.78(2.05)	0.109	0.123
Week 6	8	2.13(3.36)	1.000	9	2.78(3.11)	0.688	0.267
Week 9	8	1.13(1.81)	0.625	10	2.60(2.80)	0.203	0.306
Changes at week 6	8	0.25(3.62)		9	-0.44(2.70)		0.520
Changes at week 9	8	-0.75(2.96)		10	-1.00(2.58)		0.556
**Walking ability**							
Baseline	10	1.00(1.89)		10	3.80(3.19)		0.008*
Week 3	9	0.78(0.83)	1.000	9	3.22(3.11)	0.406	0.078
Week 6	8	1.75(2.38)	0.750	9	3.11(3.62)	0.641	0.585
Week 9	8	1.00(1.51)	0.938	10	2.70(2.45)	0.426	0.092
Changes at week 6	8	0.50(3.38)		9	0.00(3.39)		0.258
Changes at week 9	8	-0.25(2.55)		10	-1.10(3.60)		0.395
**Normal work**							
Baseline	10	1.40(2.01)		10	4.50(3.89)		0.061
Week 3	9	1.22(1.09)	1.000	9	2.56(2.60)	0.031*	0.374
Week 6	8	1.50(1.93)	0.844	9	2.67(2.96)	0.156	0.423
Week 9	8	1.13(1.25)	0.625	10	1.80(1.75)	0.070	0.457
Changes at week 6	8	-0.25(3.11)		9	-1.44(2.96)		0.309
Changes at week 9	8	-0.63(1.85)		10	-2.70(4.11)		0.189
**Relations with other people**							
Baseline	10	0.50(0.97)		10	0.50(0.85)		1.000
Week 3	9	0.67(0.71)	1.000	9	0.22(0.44)	0.500	0.253
Week 6	8	1.25(2.05)	0.750	9	0.67(1.66)	1.000	0.310
Week 9	8	0.75(1.04)	0.750	10	1.00(2.11)	0.500	0.725
Changes at week 6	8	0.63(2.07)		9	0.11(1.17)		0.660
Changes at week 9	8	0.13(1.46)		10	0.50(1.35)		0.903
**Sleep**							
Baseline	10	1.90(2.88)		10	4.30(2.16)		0.071
Week 3	9	1.00(1.12)	0.406	9	3.56(2.55)	0.516	0.014*
Week 6	8	1.75(2.87)	0.750	9	3.78(3.49)	1.000	0.157
Week 9	8	1.25(1.75)	0.625	10	2.80(2.94)	0.078	0.235
Changes at week 6	8	-0.63(3.58)		9	-0.22(2.33)		0.831
Changes at week 9	8	-1.13(3.14)		10	-1.50(2.22)		0.577
**Enjoyment of life**							
Baseline	10	1.60(2.27)		10	2.60(2.84)		0.386
Week 3	9	1.33(1.73)	0.531	9	1.44(1.67)	0.391	0.837
Week 6	8	1.88(2.90)	1.000	9	3.00(3.28)	0.563	0.366
Week 9	8	1.25(1.75)	0.688	10	2.50(2.68)	0.590	0.310
Changes at week 6	8	-0.13(3.72)		9	1.11(3.41)		0.615
Changes at week 9	8	-0.75(3.06)		10	-0.10(3.51)		0.985

Note: Data are presented as mean(S.D.).

*p < 0.05. **p < 0.01.Data are analyzed by Wilcoxon signed ranks test and Mann-Whitney U test.

Table S2. Results of mechanical touch-pressure perception thresholds at fingers and toes

**Touch-Pressure perception threshold (grams)**	**Acupuncture group**			**Sham acupuncture group**			**Mann-Whitney *U* test *p*-value**
	**N**	**Mean(SD)**	**Wilcoxon signed ranks test *p*-value**	**N**	**Mean(SD)**	**Wilcoxon signed ranks test *p*-value**	
**LHT**							
Baseline	10	1.64(1.87)		10	1.62(1.41)		0.836
Post-Tx	8	0.41(0.66)	0.055	10	1.82(2.28)	0.531	0.043*
Changes at post-Tx	8	-1.54(2.12)		10	0.20(2.51)		0.049*
**LHP**							
Baseline	10	2.60(4.69)		10	1.98(2.89)		0.440
Post-Tx	8	0.25(0.23)	0.008**	10	1.44(1.99)	0.297	0.214
Changes at post-Tx	8	-2.92(5.20)		10	-0.54(1.73)		0.325
**RHT**							
Baseline	10	1.52(1.35)		10	1.74(1.31)		0.505
Post- Tx	8	0.37(0.29)	0.008**	10	1.25(1.78)	0.102	0.158
Changes at post-Tx	8	-1.43(1.37)		10	-0.50(2.20)		0.262
**RHP**							
Baseline	10	2.50(4.53)		10	1.91(1.85)		0.540
Post- Tx	8	0.28(0.20)	0.031*	10	1.27(1.77)	0.078	0.049*
Changes at post-Tx	8	-2.75(5.06)		10	-0.64(1.13)		0.262
**LFT**							
Baseline	10	14.02(30.57)		10	10.30(17.99)		0.961
Post- Tx	8	2.50(2.49)	0.117	10	3.50(3.41)	0.219	0.498
Changes at post-Tx	8	-14.87(34.46)		10	-6.80(16.04)		0.531
**LFP**							
Baseline	10	20.66(56.17)		10	9.61(18.09)		0.492
Post- Tx	8	2.35(5.13)	0.047*	10	3.24(4.60)	0.055	0.344
Changes at post-Tx	8	-23.40(57.43)		10	-6.36(13.99)		0.880
**RFT**							
Baseline	10	12.80(17.30)		10	5.28(7.55)		0.083
Post- Tx	8	2.43(2.94)	0.039*	10	4.94(4.71)	0.922	0.194
Changes at post-Tx	8	-12.45(19.99)		10	-0.34(7.15)		0.041*
**RFP**							

Baseline	10	6.46(6.53)		10	9.36(18.01)		0.954
Post- Tx	8	2.87(5.06)	0.031*	10	3.94(3.77)	0.539	0.390
Changes at post-Tx	8	-4.93(6.11)		10	-5.41(15.95)		0.264

Note: Data are presented as mean(S.D.). *Abbreviations*: S.D., standard deviation; LHT, Left hand's middle fingertip; LHP, Left hand's palm; RHT, Right hand's middle fingertip; RHP, Right hand's palm; LFT, Left foot's big toe tip; LFP, Left foot's plantar; RFT, Right foot's big toe tip; RFP, Right foot's plantar; Tx, treatment.

*p < 0.05. **p < 0.01.Data are analyzed by Wilcoxon signed ranks test and Mann-Whitney *U* test.

Table S3. Results of patient-reported outcome in the WHOQOL-BREF(Taiwan version)

Four domains in WHOQOL-BREF	Acupuncture group			Sham acupuncture group			Two sample T test *p*-value
	N	Mean(SD)	Paired t Test *p*-Value	N	Mean(SD)	Paired t Test *p*-Value	
**Physical health**							
Baseline	10	60.80(15.97)		10	55.20(12.69)		0.397
Post-Tx	8	60.88(17.88)	0.473	10	59.50(11.34)	0.335	0.845
Changes at post-Tx	8	3.63(13.52)		10	4.30(13.36)		0.917
**Psychological**							
Baseline	10	51.40(13.79)		10	45.20(16.58)		0.375
Post-Tx	8	55.50(18.62)	0.185	10	52.50(13.52)	0.020*	0.697
Changes at post-Tx	8	6.13(11.79)		10	7.30(8.14)		0.806
**Social relationships**							
Baseline	10	62.10(9.56)		10	54.50(10.22)		0.103
Post- Tx	8	68.00(11.15)	0.085	10	58.90(10.80)	0.272	0.099
Changes at post-Tx	8	6.88(9.69)		10	4.40(11.90)		0.641
**Environment**							
Baseline	10	61.90(8.20)		10	61.40(13.02)		0.919
Post- Tx	8	61.75(10.33)	0.704	10	66.30(11.84)	0.191	0.405
Changes at post-Tx	8	-1.63(11.60)		10	4.90(10.97)		0.239

Note: Data are presented as mean(S.D.). *Abbreviations*: S.D., standard deviation; WHOQOL: World Health Organization Quality of Life Scale; Tx, treatment.

*p < 0.05. Data are analyzed by paired *t*-test and two-sample *t*-test.
